# Charcoal as a Disinfectant

**Published:** 1855-04

**Authors:** 


CHARCOAL AS A DISINFECTANT.—The *London Lancet* thus describes a simple method of obviating the unpleasant odor of sick rooms, &c., as practised in one of the hospitals in that city.

"The charcoal, as tried by Dr. Stenhouse in the dissecting rooms at St. Bartholomew's Hospital, is in rough powder in shallow tin boxes, something in a rude way like lump-sugar broken small; the boxes are about two feet long and one wide, as nearly as we can now bring them to mind. The effect, as we have been told, in removing noxious smells is quite remarkable; there is no soiling or blackness visible at all. We should say that in wards of hospitals, where there were large purulent discharges from ulcers, such boxes under the beds would be very useful."

